# Students' competencies in Problem-Based Learning influence evaluation of tutors

**DOI:** 10.3389/fpsyg.2026.1792056

**Published:** 2026-06-19

**Authors:** Lingyu Cui, Zhirong Liu, Yuanyao Cao, Rui Qiu, Yanrong Li, Guijie Ren, Zhongfa Zhang, Hua Cong

**Affiliations:** 1 Department of Pathogenic Biology, School of Basic Medicine, Cheeloo College of Medicine, Shandong University, Jinan, China; 2 Shandong Public Health Clinical Center, Medical Integration and Practice Center, Cheeloo College of Medicine, Shandong University, Jinan, China; 3 Department of Biochemistry and Molecular Biology, School of Basic Medicine, Cheeloo College of Medicine, Shandong University, Jinan, China

**Keywords:** competencies enhancement, mediation analysis, Problem-Based Learning (PBL), students' attitude, tutor evaluation

## Abstract

In Problem-Based Learning (PBL), tutors transform from traditional knowledge transmitters into learning facilitators, which renders accurate tutor evaluation essential to the effectiveness of PBL implementation. Nevertheless, it remains underexplored whether students' attitudes toward the PBL methodology exert systematic influences on tutor evaluation outcomes. To address this, a cross-sectional study conducted an online questionnaire survey among 263 medical students from Shandong University to examine the association between students' PBL attitudes and tutor evaluations, as well as the potential mediating role of perceived competency enhancement. The questionnaire collected participants' demographic information and adopted a 5-point Likert scale to assess three core variables: students' attitudes toward PBL, perceived competency enhancement, and tutor evaluation scores. Correlation analysis, hierarchical regression analysis, and structural equation modeling were employed for statistical data analysis. The results indicated that both students' PBL attitudes and perceived competency enhancement were significantly positively correlated with tutor evaluations (*P* < 0.01). After controlling for demographic variables, hierarchical regression analysis revealed that these two factors collectively explained 54.5% of the variance in tutor evaluations. Mediation analysis further verified that students' PBL attitudes could positively predict tutor evaluations through both direct and indirect pathways. The direct effect of PBL attitudes on tutor evaluations was 0.124, while perceived competency enhancement served as a significant partial mediator with an indirect effect of 0.390, accounting for 75.97% of the total effect. In conclusion, students' PBL attitudes influence their evaluations of tutors predominantly via the mediating pathway of perceived competency enhancement. Consistent with the hypothesized attributional framework, these findings suggest that PBL tutors should focus on fostering students' comprehensive competencies, so as to guarantee the validity of faculty evaluation and promote the iterative optimization of PBL teaching practice.

## 1 Introduction

Problem-Based Learning (PBL) is a student-centered pedagogical approach in which small student groups collaboratively tackle authentic clinical problems under tutor supervision (Albanese and Mitchell, 1993). Distinct from conventional lecture-based passive learning, the active inquiry framework of PBL effectively improves long-term knowledge retention while fostering core professional competencies, including clinical reasoning, independent thinking and collaborative problem-solving skills (Zhou et al., 2016; Cui et al., 2023; Ge et al., 2025). As a key component of ongoing medical education modernization reforms in China, PBL has been progressively integrated into local medical curricula (Lam et al., 2006). To date, this innovative teaching model has achieved successful implementation across multiple medical disciplines, covering physical diagnostics (Wang et al., 2016), radiology (Zhang et al., 2018), pharmacy (Zhou et al., 2016), and psychology (Gao et al., 2020).

Given the student-driven nature of PBL pedagogy, students' subjective attitudes toward this learning model serve as a critical predictor of their learning engagement and ultimate academic outcome (Chu et al., 2023; Broseghini et al., 2024). Specifically, negative attitudes toward PBL often lead to low levels of learning participation, superficial analysis of clinical problems, and excessive reliance on tutor guidance, which fundamentally undermine the core educational goals of PBL. In contrast, students with positive PBL attitudes tend to engage more actively in group discussions, achieve deeper knowledge integration, and report higher learning satisfaction (Bista et al., 2024). Since students' attitudes directly shape the proactive learning behaviors that PBL aims to cultivate, exploring the mechanism of attitudinal impact is essential for standardized and effective PBL implementation in medical education.

Beyond student-related factors, the successful delivery of PBL heavily depends on tutor performance, which involves a fundamental role transformation from a traditional knowledge transmitter to a learning facilitator, academic guide and professional advisor (Li and Chen, 2018). Unlike didactic teaching, PBL requires tutors to inspire students' critical thinking through targeted questioning, coordinate interactive group dynamics, and guide students to access and utilize learning resources independently, rather than providing direct answers or rote knowledge indoctrination (Chan, 2008; Lyberg-Åhlander et al., 2014). Despite this clear role orientation, current PBL tutor evaluation systems predominantly focus on tutors' observable teaching behaviors and objective teaching performance (Papinczak, 2010; Demirören et al., 2020), while largely neglecting the influence of students' subjective perceptions on evaluation results.

From attribution theory (Weiner, 1985), students' tutor evaluations are essentially shaped by their subjective causal reasoning regarding personal learning gains. Students commonly attribute their perceived improvement in competency and learning progress to high-quality tutor guidance, thereby leading to more favorable evaluation scores (Cohen, 1981; Theall and Franklin, 2001). Such subjective evaluation bias is often overlooked in existing assessment frameworks, which may lead to unfair assessment of competent tutors and misguide the directional optimization of PBL teaching and tutor training. To address this research gap and improve the validity of faculty evaluation in PBL teaching, this cross-sectional study aims to clarify the underlying psychological mechanisms linking these key influencing variables.

This study primarily explores the mediating effect of perceived competency enhancement on the association between medical students' PBL attitudes and their subjective tutor evaluations. The findings of this study will reveal psychological pathways by which student attitudes and perceived competency enhancement influence tutor assessment. Furthermore, this research provides empirical insights and practical suggestions to optimize tutor training systems and improve the scientific rigor and validity of PBL tutor evaluation in medical education.

## 2 Materials and methods

### 2.1 Study design and participants

A cross-sectional study was conducted between July and August 2024. Participants were recruited from Cheeloo College of Medicine, Shandong University. The college adopts a well-established problem-based learning curriculum in foundational medical disciplines, including physiology and pharmacology. Most participants were Clinical Medicine undergraduates, while the rest came from non-clinical majors: Public Health and Preventive Medicine, Pharmaceutical Science, Nursing, Biomedical Sciences, and others.

### 2.2 Data collection

The questionnaire was designed mainly based on previous related studies (Rimal et al., 2015; Mal et al., 2021). It comprised four sections: demographic information, students' attitudes to PBL, competencies enhancement and students' evaluation of tutor. Each section consisted of several corresponding items. Design details of questionnaire are shown in Table 1. Subsequent sections elaborate on specific questions presented as single-choice questions and five-point Likert scale items.

**Table 1:** Research questionnaire sections and items.

Section heading	Items	Measure	References
Your Demographics	Grade, gender, age, major, academic ranking	Box	
	Time spent (outside of class)	Box	
Attitude to PBL	Att1: learning process	5-point Likert scale	Rimal et al., 2015; Mal et al., 2021
	Att2: learning effectiveness		
	Att3: learning interest		
Competencies enhancement	Com1: comprehension ability	5-point Likert scale	Rimal et al., 2015
	Com2: critical thinking skills		
	Com3: presentation skills		
	Com4: teamwork skills		
	Com5: peer learning ability		
Evaluation of tutor	Eva1: emotional engagement	5-point Likert scale	Rimal et al., 2015; Mal et al., 2021
	Eva2: guidance strategies		
	Eva3: classroom management		

Participants completed an anonymous electronic questionnaire on the Wenjuanxing platform. Of the 291 collected responses, 263 were retained as valid after excluding those with irregular completion times or missing items. Complete-case (listwise deletion) analysis was applied to this final dataset, with all data anonymized for confidentiality.

### 2.3 Statistical analysis

Most statistical analyses were conducted using IBM SPSS version 27.0 (with the Process plugin version 5.0) and AMOS version 24.0. *P* < 0.05 (two-sided test) was considered statistically significant. Demographic characteristics were summarized as mean ± standard deviation (SD) or median (Inter-Quartile Range [IQR]), and frequencies (%) for categorical data. Subsequent analyses involved assessing reliability and validity tests, difference analyses, correlation analyses and regression analyses. Finally, structural equation modeling (SEM) was employed to test the hypothesized mediation model, where competencies enhancement mediates the path between students' attitude to PBL and their evaluation of tutor.

Cronbach's alpha coefficient is widely used to assess questionnaire reliability, with values above 0.90 considered “excellent” (George and Mallery, 2024). Confirmatory factor analysis (CFA) evaluated the construct validity, with the Kaiser-Meyer-Olkin (KMO) test and Bartlett's test of sphericity assessing the data's suitability for factor analysis. A KMO value exceeding 0.80 signifies a clear and stable factorial structure, and a statistically significant Bartlett's test (*P* < 0.05) confirms sufficient correlations among variables, thereby justifying proceeding with the analysis. Construct validity was further assessed by examining the average variance extracted (AVE) and composite reliability (CR), both of which should exceed 0.5 (Berger, 2021). Bootstrap tests (2,000 repetitions) evaluated mediation significance using 95% bias-corrected confidence intervals. Model fit was evaluated according to these criteria: a chi-squared minimum discrepancy divided by degrees of freedom (CMIN/DF) below 5 is acceptable, while the comparative fit index (CFI), Tucker-Lewis index (TLI), incremental fit index (IFI), and goodness of fit index (GFI) should all reach 0.90 or higher. The root mean square error of approximation (RMSEA) should be 0.08 or lower (Sathyanarayana and Mohanasundaram, 2024). Furthermore, construct validity was considered satisfactory as all factor loadings exceeded the recommended threshold of 0.70 (Fornell and Larcker, 1981).

## 3 Results

### 3.1 Descriptive analysis

A total of 263 medical students were enrolled in the study, including 46.0% males and 54.0% females. Of all participants, 82.5% majored in Clinical Medicine, and most were sophomores (30.0%) and juniors (26.6%). In terms of academic performance ranking, 41.8% of students fell within the 20%-50% percentile range. Most respondents reported spending 2–4 h (35.4%) and 4–8 h (34.6%) weekly on extracurricular PBL learning. Further demographic characteristics are presented in Supplementary Table S1.

**Figure 1:**
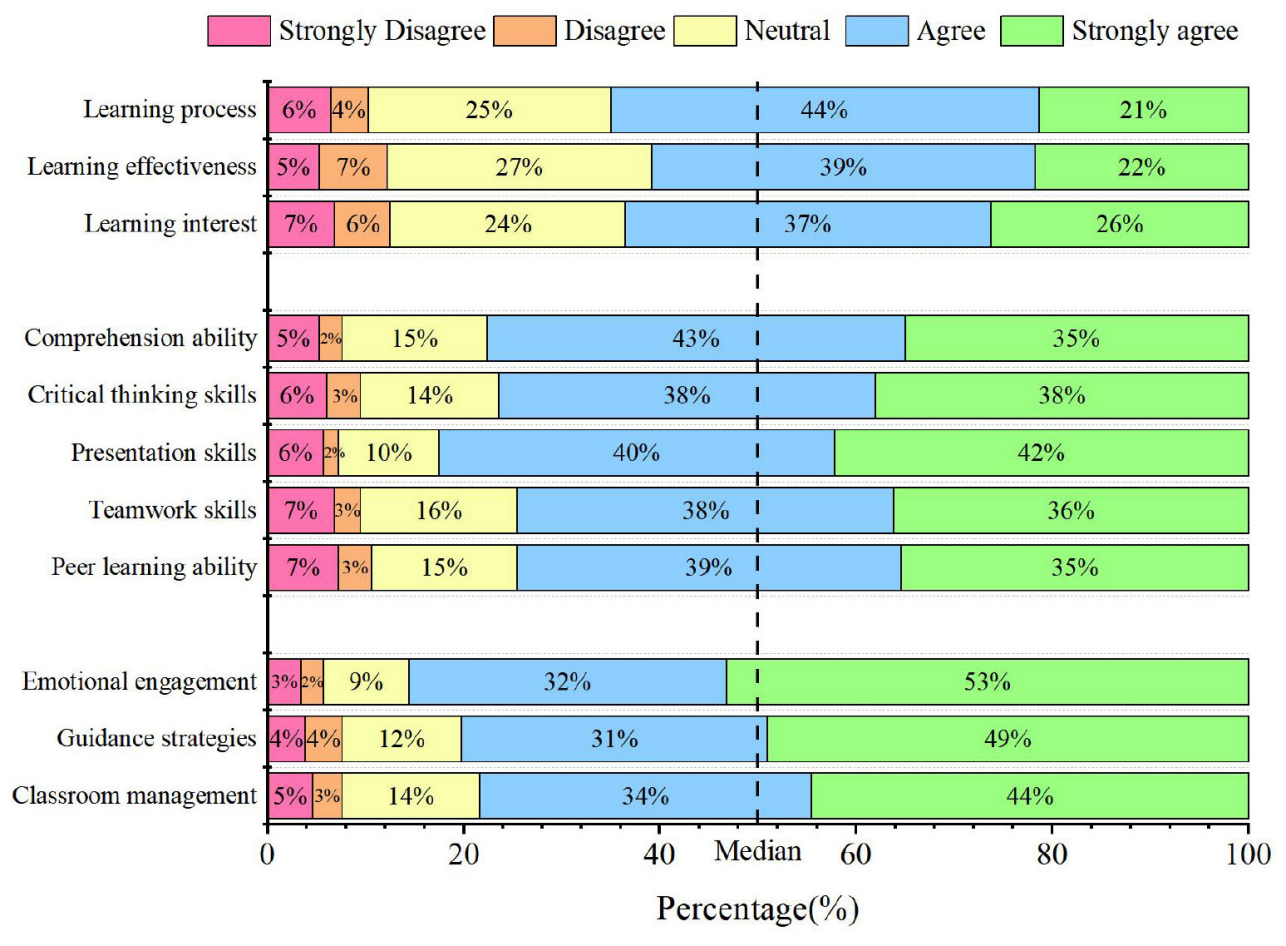
Distribution of responses to Likert-scale items on attitude to PBL, competencies enhancement, and evaluation of tutor.

As shown in Figure 1, student perceptions were positive across all three surveyed domains: attitude toward PBL, evaluation of tutor, and perceived competency enhancement. Tutor evaluations were rated notably high, particularly for tutors' enthusiasm and emotional engagement, which received a median score of 5 (“Strongly Agree”). Students also demonstrated acceptance of the PBL model, with median responses in the “Agree” range. Detailed responses are provided in Supplementary Table S2. Furthermore, independent-samples *t*-tests revealed significant demographic differences. Female students reported significantly more favorable attitudes toward PBL (M ± SD: 3.80 ± 0.92 vs. 3.54 ± 1.15) and higher competency development ratings (4.11 ± 0.94 vs. 3.85 ± 1.10) compared to male students (shown in Supplementary Table S3).

### 3.2 Reliability, validity, and confirmatory factor analysis

The measurement model demonstrated satisfactory reliability and validity. Reliability was supported by high Cronbach's alpha coefficients (>0.9) for all three dimensions. Validity was confirmed by a KMO test of 0.922 (*P* < 0.001) and adequate fit indices (χ^2^/df = 2.614; GFI = 0.932; TLI = 0.976; CFI = 0.982; IFI = 0.982; NFI = 0.972; RMSEA = 0.078). All observed variables presented in Table 2 demonstrated standardized factor loadings ranging from 0.885 to 0.959 (all >0.70, *P* < 0.001), confirming effective item measurement of latent variables. Both CR values (0.949–0.967) and AVE values (0.855–0.893) surpassed 0.50, respectively, indicating excellent internal consistency and convergent validity. Discriminant validity was also established, as all AVE values surpassed their corresponding maximum shared squared variance (MSV) and average shared squared variance (ASV) (Cheung et al., 2024).

**Table 2:** Reflective constructs assessment.

Construct	Factor loading	α	AVE	CR	MSV	ASV
Att1	0.946	0.961	0.893	0.962	0.429	0.366
Att2	0.946					
Att3	0.943					
Eva1	0.885	0.947	0.86	0.949	0.524	0.413
Eva2	0.959					
Eva3	0.937					
Com1	0.943	0.967	0.855	0.967	0.524	0.477
Com2	0.93					
Com3	0.934					
Com4	0.915					
Com5	0.901					

α, Cronbach's Alpha Coefficient; CR, Composite reliability; AVE, Average variance extracted; MSV, Maximum shared squared variance; ASV, Average shared squared variance; Att, Attitude to PBL; Eva, Evaluation of tutor; Com, Competencies enhancement.

### 3.3 Pearson correlation analysis

To investigate correlations among variables, we employed Pearson's correlation coefficient analysis. The results shown in Supplementary Table S4 indicate that students' attitude to PBL, evaluation of tutor, and competencies enhancement all exhibit significant positive correlations with each other (*P* < 0.01).

### 3.4 Hierarchical regression analysis of tutor evaluation

Hierarchical regression was performed to assess how students' attitude to PBL and competencies enhancement influenced tutor evaluation, after controlling for demographic variables. In Model 1, demographic variables (grade, gender, major, and academic ranking) were included. Model 2 added the two key predictors: attitude toward PBL and competency enhancement. The results shown in Table 3 indicate that the model met key prerequisite assumptions: no multicollinearity existed [Variance Inflation Factor (VIF) values: 1.004–1.797, all < 5], and residuals were independent (Durbin-Watson test value = 2.110), ensuring the reliability of subsequent results. The results showed that in Model 2, students' attitude to PBL and the competencies enhancement explained 54.5% of the variance in tutor evaluations; after controlling for major's influence, the enhancement of competencies showed a positive relationship with the evaluation of tutor (standardized regression coefficient = 0.639, *P* < 0.001).

**Table 3:** The hierarchical regression results of tutor evaluation's predictors (*N* = 263).

Variables	Model 1	Model 2
	β	β
Grade	0.079	0.043
Major	−0.036	−0.092^*^
Gender	0.106	0.02
Academic ranking	−0.142^*^	−0.034
Attitude to PBL	/	0.129^*^
Competencies enhancement	/	0.639^***^
*R^2^*	0.039	0.545
Δ*R^2^*	0.039	0.506

^*^P < 0.05.

^**^P < 0.01.

^***^P < 0.001.

β, standardized regression coefficient. **Model 1** included demographic variables (grade, gender, major, academic ranking). **Model 2** included the variables in Model 1, in addition to students' attitude to PBL and competencies enhancement. *R*^2^, cumulative explained variance; Δ*R*^2^, change in *R*^2^.

### 3.5 Mediation analysis within structural equation modeling

**Figure 2:**
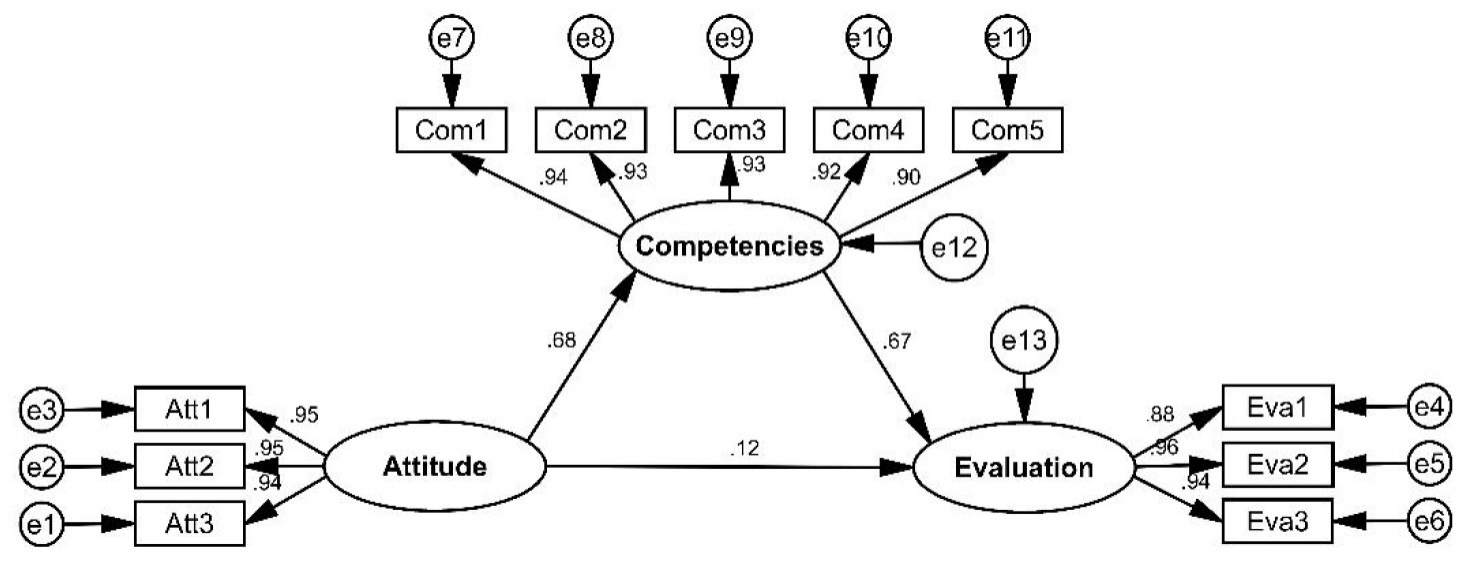
Mediation effects of competencies enhancement on attitude to PBL and evaluation of tutor coefficients. Att (Attitude), Attitude to PBL; Com (Competencies), Competencies Enhancement; Eva (Evaluation), Evaluation of Tutor; Values in the graph are standardized regression coefficients.

To more flexibly analyze the potential relationships among students' attitudes toward PBL, competencies enhancement, and tutor evaluation, we conducted a mediation analysis using SEM (Figure 2). The model fit indices were excellent (χ^2^/df = 2.614, RMSEA = 0.078, GFI = 0.932, TLI = 0.976, IFI = 0.982, NFI = 0.972), indicating a good fit of the SEM.

To test the mediation model proposed in this study, we analyzed the mediating effect using the Bootstrap method. Results (see Supplementary Table S5 for details) indicate that the overall effect of students' attitude to PBL on evaluation of tutor is significant [Effect = 0.514, 95% CI (0.419, 0.609), *P* < 0.001]; the direct effect remained significant after controlling for the mediating variable of personal competency development [Effect = 0.124, Effect proportion = 24.03%, 95% CI (0.020, 0.227), *P* < 0.05]; while the indirect effect through the improvement of competencies was 0.390 (Effect proportion = 75.97%), with its Bootstrap 95% confidence interval (0.246, 0.523) not containing zero, indicating a significant indirect effect. These findings demonstrate that students' perceived competencies enhancement serves as a partial mediator in the relationship between their attitudes toward PBL and evaluations of tutors' performance.

## 4 Discussion

This study identified a notable mediating mechanism underlying the relationship between students' attitudes toward PBL and their evaluations of tutors. Mediation analysis is a widely adopted method in medical education research to explore the interactive pathways between variables (Zhang et al., 2024; Meng et al., 2025). While students' positive attitudes directly contribute to satisfactory tutor evaluations, 75.97% of such influence takes effect indirectly via perceived competency improvement, covering critical thinking, teamwork, presentation skills, comprehension, and peer learning abilities. When examining the specific items of the tutor evaluation dimension, students scored tutors remarkably high on emotional engagement, with a median score of 5. Guidance strategies and classroom management also attained favorable median scores of 4 each. These findings reflect the sound interpersonal atmosphere and positive emotional rapport built by tutors throughout PBL activities.

The direct relationship between students' attitudes toward PBL and tutor evaluation (24% of the total effect) might be explained by the halo effect (Huang et al., 2023). Driven by this cognitive bias, students who favor the PBL methodology may project their enthusiasm onto the tutor, rating them highly regardless of specific pedagogical actions. While such subjective attitudinal filters are well documented in general teaching evaluations (Cannon and Cipriani, 2022), existing PBL research has predominantly focused on how tutor performance impacts student learning engagement (Yun et al., 2023; Kassab et al., 2024). Our findings provide a complementary perspective by showing that students' evaluations are not solely driven by the tutor's actual performance. Instead, these evaluations strongly reflect the subjective, attitudinal lens through which students view the PBL methodology.

However, the dominance of the indirect pathway (75.97% of total effect) indicates that the halo effect alone provides an incomplete explanation. To understand this indirect pathway, we draw upon attribution theory as a potential interpretive framework. According to Heider's attribution theory, individuals categorize the causes of their behavior into dispositional (internal) and situational (external) factors (Solomon, 1978). When considering personal achievement, people focus primarily on ability and effort (McClure et al., 2011; Graham, 2020). In many areas of medicine, numerous behavioral outcomes are more or less governed by attributive thinking (Liu et al., 2025; Song et al., 2025; Xu et al., 2025; Qiu et al., 2025). Within PBL contexts, when students perceive tangible improvements in their core competencies, they tend to attribute these gains directly to the tutor's strategic facilitation and scaffolding. Therefore, positive attitudes toward PBL drive greater learning investment, generating perceived skill gains that are ultimately credited to the tutor. This attributional process offers a possible explanation for why competencies enhancement strongly mediates the relationship between students' attitude and their evaluation of tutors.

The results of the study have practical implications for medical education: institutions should make efforts to enhance students' pedagogical acceptance, while simultaneously training tutors to prioritize cultivating students' specific competencies rather than solely building affective rapport. By clarifying the link between tutor performance and students' learning outcomes, faculty can ensure evaluations reflect true pedagogical effectiveness rather than attitudinal confounds. This transparency ultimately fosters more valid assessment systems and drives continuous improvement in PBL implementation.

## 5 Limitations and future directions

While this study offers valuable insights into PBL tutor evaluations, it still has several limitations. First, the cross-sectional, single-institution design limits causal inference and generalizability. Future research should validate the mediation model using multi-institutional samples with longitudinal designs to establish causality and trace developmental trajectories across different educational environments. Second, treating “competencies enhancement” as a unitary construct may obscure internal heterogeneity; subsequent studies should employ subscales to clarify distinct mediating pathways. Finally, reliance on self-reported data can introduce common method variance (CMV), which can inflate empirical correlations. To mitigate this, future investigations would benefit from incorporating multi-informant approaches or objective performance metrics.

## 6 Conclusion

This study verified a mediation model indicating that students' attitudes toward PBL affect their tutor assessments predominantly through perceived competencies enhancement rather than direct attitudinal transfer. This pattern implies that positive evaluations arise from attribution judgment, where students attribute tangible skill advancement to tutors rather than being influenced merely by the halo effect. These findings suggest that tutor training should focus on fostering students' specific capabilities. Such practice enables evaluation systems to truly reflect teaching quality, facilitating credible faculty assessment and sustained optimization of PBL teaching.

## Data Availability

The original contributions presented in the study are included in the article/Supplementary material, further inquiries can be directed to the corresponding authors.
