# Supplementary material for: Students' competencies in Problem-Based Learning influence evaluation of tutors
Source: Front Psychol. 2026 Jun 19;17:1792056. doi: 10.3389/fpsyg.2026.1792056 (PMC13328494; doi:10.3389/fpsyg.2026.1792056)
Supplement: Supplementary file 1 [file Supplementary_file_1.docx]

**Supplementary Table**

**Students’ Competencies in Problem-Based Learning Influence Evaluation of Tutor**

**Table S1** Demographic and scholarly characteristics of study participants

| **Demographic and scholarly characteristics** | | **Number (%)** |
| --- | --- | --- |
| **Gender** | Male | 121(46%) |
|  | Female | 142(54%) |
| **Grade** | 1 | 34(12.9%) |
|  | 2 | 79(30%) |
|  | 3 | 70(26.6%) |
|  | 4 | 35(13.3%) |
|  | 5 | 45(17.1%) |
| **Major** | Clinical | 217(82.5%) |
|  | Other Medical Specialities | 46(17.5%) |
| **Academic ranking** | 20% | 76(28.9%) |
|  | 20%-50% | 110(41.8%) |
|  | 50%-100% | 77(29.3%) |
| **Time spent (outside of class)** | <2h per week | 33(12.5%) |
|  | 2-4h per week | 93(35.4%) |
|  | 4-8h per week | 91(34.6%) |
|  | >8h per week | 46(17.5%) |

Institutional identifiers have been removed to maintain anonymity for peer review.

**Table S2** Descriptions of variables or indicators

| **Variable/indicator** | | **Median (IQR)** |
| --- | --- | --- |
| **Attitude**  **towards PBL** | 1.Learning process | 4(3,4) |
|  | 2.Learning effectiveness | 4(3,4) |
|  | 3.Learning interest | 4(3,5) |
| **Evaluation of tutor** | 1.Emotional engagement | 5(4,5) |
|  | 2.Guidance strategies | 4(4,5) |
|  | 3.Classroom management | 4(4,5) |
| **Competencies enhancement** | 1.Comprehension ability | 4(4,5) |
|  | 2.Critical thinking skills | 4(4,5) |
|  | 3.Presentation skills | 4(4,5) |
|  | 4.Teamwork skills | 4(3,5) |
|  | 5.Peer learning ability | 4(3,5) |

Institutional identifiers have been removed to maintain anonymity for peer review.

**Table S3** Mean Scores, Standard Deviations, and T-tests for Three Sections by Gender

| **Section** | **Gender**  **Male (n = 121) Female (n = 142)** | | **t** |
| --- | --- | --- | --- |
| **Attitude towards PBL** | 3.54 ± 1.15 | 3.80 ± 0.92 | -2.002* |
| **Competencies enhancement** | 3.85 ± 1.10 | 4.11 ± 0.94 | -2.069* |
| **Tutor evaluation** | 4.08 ± 1.00 | 4.29 ± 0.94 | -1.722 |

*, *P*< 0.05, institutional identifiers have been removed to maintain anonymity for peer review.

**Table S4** Correlation coefficients matrix of variables

| **Variable/Indicator** | **1** | **2** | **3** |
| --- | --- | --- | --- |
| **1.Attitude towards PBL** | 1 |  |  |
| **2.Evaluation of Tutor** | .550** | 1 |  |
| **3.Competencies enhancement** | .655** | .724** | 1 |

**, *P* < 0.01, institutional identifiers have been removed to maintain anonymity for peer review.

**Table S5** Total, direct, and indirect effects of the mediator model(N=263)

| **Model** | **Effect** | **SE** | **t** | **P** | **95% BC CI** | **Effect proportion** |
| --- | --- | --- | --- | --- | --- | --- |
| **Total effect of students’ attitude →Evaluation of Tutor** | 0.514 | 0.048 | 10.64 | <0.001 | [0.419,0.609] |  |
| **Direct Effect of Attitude towards PBL → Evaluation of Tutor** | 0.124 | 0.052 | 2.36 | 0.019 | [0.020,0.227] | 24.03% |
| **Indirect Effect via Skills and Competencies Enhancement** | 0.390 | 0.071 |  |  | [0.246,0.523] | 75.97% |

BC, bias-corrected; CI, confidence interval; SE, standard error; Institutional identifiers have been removed to maintain anonymity for peer review.
